# Supplementary material for: Microorganisms in Macroalgae Cultivation Ecosystems: A Systematic Review and Future Prospects Based on Bibliometric Analysis
Source: Microorganisms. 2025 May 12;13(5):1110. doi: 10.3390/microorganisms13051110 (PMC12114298; doi:10.3390/microorganisms13051110)
Supplement: Supplementary file 1 [file microorganisms-13-01110-s001.zip › microorganisms-3592012-supplementary.pdf]

Microorganisms in Macroalgae Cultivation Ecosystems: A Systematic Review and Future Prospects Based on Bibliometric Analysis

Yinglong Chen[1,#], Pengbing Pei[1,3,#], Muhammad Aslam[1,5], Muhamad Syaifudin[1], Ran Bi[1,3], Ping Li[1,4], Hong Du[1,2,*]

[1] Guangdong Provincial Key Laboratory of Marine Biotechnology, College of Science, Shantou University, Shantou 515063, China.

[2] Shantou University-Università Politecnica delle Marche (STU-UNIVPM) Joint Algal Research Center, College of Science, Shantou University, Shantou 515063, China.

[3] Guangdong Provincial Key Laboratory of Marine Disaster Prediction and Prevention, College of Science, Shantou University, Shantou 515063, China.

[4] Guangdong Engineering Technology Research Center of Offshore Environmental Pollution Control, Shantou University, Shantou 515063, China.

[5] Faculty of Basic Sciences, Bolan University of Medical and Health Sciences, Quetta, Pakistan.

* Corresponding author: Hong Du, hdu@stu.edu.cn

[#] Both authors contributed equally to the article.

Retrieval methodology

The retrieval terms were TS= (("macroalgae" NOT "feed" NOT "gut" NOT ferment* NOT "reactor" NOT "fuel") AND ("*cultivation*" OR "*farm*" OR "*culture*") OR (("seaweed*" OR "laminaria japonica*" OR "kelp" NOT "feed" NOT gut NOT ferment* NOT "reactor" NOT "fuel") AND ("*cultivation*" OR "*farm*" OR "*culture*")) OR (("gelidium amansii*" NOT "agar plate*" NOT "agar dilution" NOT "culture media" NOT "feed" NOT "gut" NOT "ferment*" NOT "reactor" NOT "fuel") AND ("*cultivation*" OR "*farm*" OR "*culture*")) OR (("laver*" OR "porphyra*" NOT "feed" NOT "gut" NOT "ferment*" NOT "reactor" NOT "fuel") AND ("*cultivation*" OR "*farm*" OR "*culture*")) OR (("pelvelia siliguosa*" OR "carrageen*" NOT "feed" NOT "gut" NOT "ferment*" NOT "reactor" NOT "fuel") AND ("*cultivation*" OR "*farm*" OR "*culture*")) OR (("sargasso*" OR "carnage*" NOT "feed" NOT "gut" NOT "ferment*" NOT "reactor" NOT "fuel") AND ("*cultivation*" OR "*farm*" OR "*culture*")) OR (("undaria*" NOT "feed" NOT "gut" NOT "ferment*" NOT "reactor" NOT "fuel") AND ("*cultivation*" OR "*farm*" OR "*culture*")) OR (("ulva*" NOT "feed" NOT "gut" NOT "ferment*" NOT "reactor" NOT "fuel") AND ("*cultivation*" OR "*farm*" OR "*culture*")) OR (("gracilaria*" NOT "feed" NOT "gut" NOT "ferment*" NOT "reactor" NOT "fuel") AND ("*cultivation*" OR "*farm*" OR "*culture*"))) AND TS = ("microorganism*" OR "microbio*" OR "microbial*" OR "bacteria*").

The asterisk (*) in search process was used to retrieve all potential derivatives of the words, while the quote marks are used to obtain accurate and exact formulations. As a result, "ferment*" serves as the root phrase for other words, including “fermentation”, “fermentative”.

Boolean operators AND, OR, and NOT are logical connectors used to systematically combine or exclude keywords in database searches, refining the scope of literature retrieval by intersecting (AND), broadening (OR), or restricting (NOT) results. TS represented the "theme subject" search in the WoS database.

Table S1. Main information about bibliometric analysis data

Items	Results
Timespan	2003-2023
Sources (Journals, Books, etc)	220
Documents	610
Imported article	543
Imported review	67
Annual Growth Rate（%）	11.46
Document Average Age	8.14
Average citations per doc	35.08
References	28874
Keywords Plus (ID)	2339
Author's Keywords (DE)	1933
Authors	2516
Co-Authors per Doc	5.25
International co-authorships (%)	28.69
Corresponding Countries	51
Institution	716

Table. S2. Top 25 institutions by publications number

Affiliation	Publications number
Chinese Academy of Sciences	62
Centre National de la Recherche Scientifique (CNRS)	53
Sorbonne Université	40
Ocean University of China	40
Japan Fisheries Research and Education Agency (FRA)	30
Indian Council of Agricultural Research (ICAR)	28
University of California System	26
University of New South Wales Sydney	25
Helmholtz Association	25
Ningbo University	22
Ghent University	22
Institute of Oceanology, CAS	21
Universidade do Porto	20
Friedrich Schiller University of Jena	20
Laoshan Laboratory	18
Chinese Academy of Fishery Sciences	18
Xiamen University	16
Ministry of Agriculture and Rural Affairs	16
Shanghai Ocean University	15
Oregon State University	15
ICAR - Central Marine Fisheries Research Institute	15
Consejo Superior de Investigaciones Científicas (CSIC)	14
University of California, Santa Barbara	13

Yellow Sea Fisheries Research Institute, CAFS	12
University of Chinese Academy of Sciences, CAS	12

Table. S3. Top 10 authors by publications number

Authors	Publications number
Thomas Wichard	13
Jie Li	10
Stephen J. Giovannoni	9
Kajal Chakraborty	9
Suhelen Egan	9
Gaoge Wang	9
Craig A Carlson	8
Olivier De Clerck	8
Florian Weinberger	8
Haimin Chen	8

Table. S4. Journals with an publications number of 10 or Greater

Element	h index	Total Citations	Publications number
Journal of Applied Phycology	17	1234	41
Aquaculture	15	1005	29
Frontiers in Microbiology	16	727	22
Applied and Environmental Microbiology	18	3868	20
Frontiers in Marine Science	7	173	19
Environmental Microbiology	15	959	16
Antonie van Leeuwenhoek International Journal of General and Molecular Microbiology	7	144	11
Aquatic Microbial Ecology	10	505	10
FEMS Microbiology Ecology	10	447	10
Algal Research-Biomass Biofuels and Bioproducts	7	210	10
Marine Pollution Bulletin	5	122	10
